# Chicoric Acid Prevents Neuroinflammation and Neurodegeneration in a Mouse Parkinson’s Disease Model: Immune Response and Transcriptome Profile of the Spleen and Colon

**DOI:** 10.3390/ijms23042031

**Published:** 2022-02-12

**Authors:** Ning Wang, Rui Li, Bainian Feng, Yuliang Cheng, Yahui Guo, He Qian

**Affiliations:** 1 School of Food Science and Technology, Jiangnan University, Wuxi 214122, China; wangyuning31@163.com (N.W.); wxfoodcyl@126.com (Y.C.); 2 Cedars-Sinai Medical Center, Los Angeles, CA 90048, USA; lr2008525@163.com; 3 School of Pharmaceutical Science, Jiangnan University, Wuxi 214122, China; fengbainian@jiangnan.edu.cn

**Keywords:** chicoric acid, Parkinson’s disease, neuroinflammation, neurodegeneration, peripheral immune system, spleen, gut

## Abstract

Chicoric acid (CA), a polyphenolic acid compound extracted from chicory and echinacea, possesses antiviral, antioxidative and anti-inflammatory activities. Growing evidence supports the pivotal roles of brain–spleen and brain–gut axes in neurodegenerative diseases, including Parkinson’s disease (PD), and the immune response of the spleen and colon is always the active participant in the pathogenesis and development of PD. In this study, we observe that CA prevented dopaminergic neuronal lesions, motor deficits and glial activation in PD mice, along with the increment in striatal brain-derived neurotrophic factor (BDNF), dopamine (DA) and 5-hydroxyindoleacetic acid (5-HT). Furthermore, CA reversed the level of interleukin-17(IL-17), interferon-gamma (IFN-γ) and transforming growth factor-beta (TGF-β) of PD mice, implicating its regulatory effect on the immunological response of spleen and colon. Transcriptome analysis revealed that 22 genes in the spleen (21 upregulated and 1 downregulated) and 306 genes (190 upregulated and 116 downregulated) in the colon were significantly differentially expressed in CA-pretreated mice. These genes were functionally annotated with GSEA, GO and KEGG pathway enrichment, providing the potential target genes and molecular biological mechanisms for the modulation of CA on the spleen and gut in PD. Remarkably, CA restored some gene expressions to normal level. Our results highlighted that the neuroprotection of CA might be associated with the manipulation of CA on brain–spleen and brain–gut axes in PD.

## 1. Introduction

Parkinson’s disease (PD) is the most prevalent neurodegenerative movement disorder caused by the progressive loss of dopaminergic neurons in the substantia nigra pars compacta region of the midbrain [1,2]. Neuroinflammation is well documented as the common pathological characteristic of PD. Notably, the neuroinflammatory phenotypes of PD can be modulated by peripheral immunoreactions through the molecular crosstalk between resident and blood-derived cellular components, suggesting that peripheral immunity is an active participant in the neuroinflammatory and neurodegenerative progression of PD [3,4,5].

The spleen and gut, two of the largest peripheral immunological organs, were proposed as the targets for anti-neurodegeneration owing to their modulatory roles in the immune system [4,6]. For example, increased splenic macrophages with activated M1 subtype could induce the systemic proinflammatory response, leading to the neurological damage and motor disorder in a mouse model of parkinsonism [7]. In addition, the 1-methyl-4-phenyl-1,2,3,6-tetrathydropyridine (MPTP)-induced mice treated with an immune modulator exhibited increased splenocytes and spleen size, which suppressed the neuroinflammatory response, motor dysfunctions and dopaminergic neuronal depletion, implicating a mediating role of the spleen in the immunological communication between central nervous system (CNS) and peripheral immune system of PD [8,9,10]. Increasing evidence has shown that gut microbial dysbiosis might be a major mediator for neuroinflammation in PD via gut microbial metabolites through the microbiota–gut–brain axis [11,12]. Microbial-dysbiosis-driven inflammation in the gut may lead to the hyperpermeability of the colon responsible for the leaky gut and the release of gut-derived toxins in PD [13]. Moreover, α-synuclein, a neuroinflammatory mediator in PD, might retrograde transport from the enteric nervous system to the CNS [12,14], suggesting that the gut plays a key role in maintaining the balance of the gut–brain axis in PD. Taken together, the spleen and gut are the vital peripheral organs that are essential for systemic immunity, are heavily involved in the pathomechanism and development of PD, and might be the potential targets aiming to combat PD neurodegeneration.

Chicoric acid (CA), a polyphenolic acid compound obtained from plants, such as chicory, purple coneflower, lettuce, dandelion, and other edible plants, exhibits antiviral, antioxidative and anti-inflammatory activities [15]. Previous studies reported that CA possessed anti-neuroinflammatory and immunoregulatory properties [16,17,18]. However, limited researches focus on the neuroprotective effects of CA on PD, and the evidence for the influence of CA on peripheral immune organs, such as the spleen and colon, in MPTP-intoxicated PD mice is lacking.

In this paper, we demonstrate that CA prevents the neurodegenerative progression in MPTP-induced PD mice. The mechanism exploration indicates the CA-regulated immune response as well as gene expression of the spleen and colon in PD. Moreover, target genes and molecular biological mechanisms for the modulation of CA on the spleen and gut in PD are obtained by transcriptome analysis. Our study provides the evidence that the neurorescue effects of CA are associated with the peripheral immune system in PD, especially the spleen and colon, which may present the potential target organs for PD therapy.

## 2. Results

### 2.1. CA Improves Motor Deficits in PD Mice

**Figure 1:**
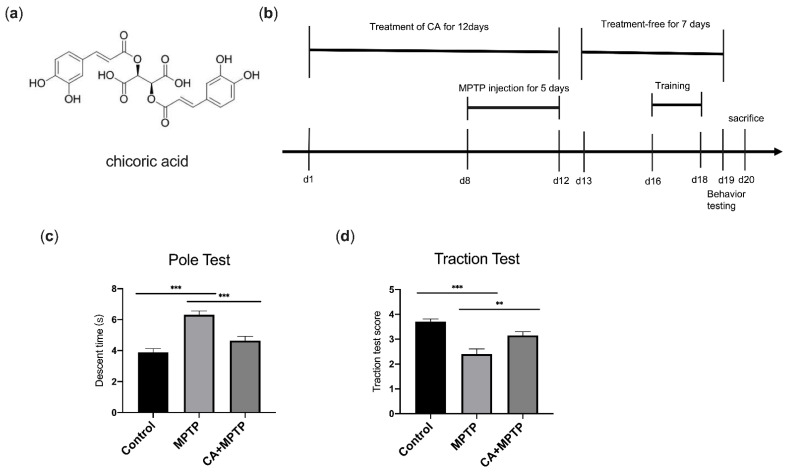
CA improved behavioral deficits in PD mice. (**a**) Chemical structure of CA. (**b**) Timeline for the experimental procedure. (**c**) Pole test: time to descend the pole. (**d**) Traction test: score of traction reflexes. Statistical comparison by one-way ANOVA with Tukey’s post hoc test. Data represent means ± SEM. ** *p* < 0.01, *** *p* < 0.001. *n* = 10.

The pole test and traction test were conducted to assess the effects of CA on motor disorder caused by MPTP. Mice treated with MPTP displayed motor dysfunction including longer pole descent time in pole test (Figure 1c) and lower scores in traction test (Figure 1d). However, CA alleviated motor disorder significantly. Mice pretreated with CA shortened the time of downward climbing from top to the bottom of the pole in the pole test (Figure 1c) and exhibited a better performance with a higher score in the measurement of gripping the rope in the traction test (Figure 1d). The results revealed that CA possessed neuroprotective effects on the motor function of PD mice.

### 2.2. CA Promoted Dopaminergic Neuron Survival and Striatal TH Levels in PD Mice

**Figure 2:**
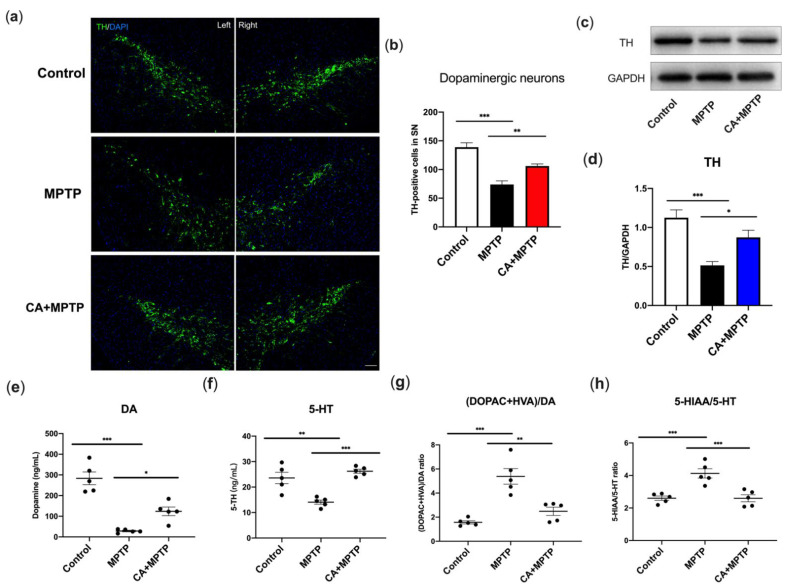
CA promoted the dopaminergic neuron survival, TH expression in striatum and the enhancement in neurotransmitters. (**a**) Representative IF staining for TH in the right and left SN, respectively. Scale bar: 100 µm. (**b**) Quantification for the number of TH-positive cells in left SN. (**c**) Representative bands of Western blotting for striatal TH expression. (**d**) Quantification for striatal TH expression, band intensity normalized to GAPDH. (**e**) The level of striatal DA. (**f**) The level of 5-TH. (**g**) The turnover rate of striatal DA ([DOPAC+HVA]/DA). (**h**) The turnover rate of striatal 5-HT (5-HIAA/5-HT). Statistical comparison by one-way ANOVA with Tukey’s post hoc test. Data represent means ± SEM. * *p* < 0.05, ** *p* < 0.01, *** *p* < 0.001. *n* = 4 for IF, *n* = 5 for the measurement of neurotransmitter and Western blotting.

The most prominent pathological feature of PD is the death of nigrostriatal dopaminergic (DA) neurons. Therefore, the survival of DA neurons in the substantia nigra (SN) was investigated by immunofluorescence (IF) staining, and the expression of striatal tyrosine hydroxylase (TH) was determined by Western blot. The results displayed that MPTP induced a decrease of TH-positive DA neurons in SN and CA pretreatment reversed the MPTP-mediated loss of DA neurons (Figure 2a,b). Western blotting analysis showed a lower expression of striatal TH in MPTP group, but CA exhibited an enhanced effect on striatal TH expression (Figure 2c,d). The above results indicated that MPTP destroyed DA neurons in SN and TH in striatum, while this lesion was attenuated by CA.

### 2.3. CA Improved the Reduction in Striatal Dopamine and Serotonin of PD Mice

Striatal neurotransmitters, dopamine (DA) and 5-hydroxytryptamine (5-HT), and their metabolites, including 3,4-dihydroxyphenylacetic (DOPAC), homovanillic acid (HVA) and 5-hydroxyindoleacetic acid (5-HIAA), were detected by high-performance liquid chromatography (HPLC) with fluorescence detection. The turnover of DA and 5-HT was used to evaluate their metabolism in PD represented as the ratio of (DOPAC+HVA)/DA and 5-HIAA/5-HT, respectively. As shown in Figure 2e–h, MPTP mice displayed a decrease of DA and 5-HT, as well as an increased ratio of (DOPAC+HVA)/DA and 5-HIAA/5-HT. CA increased the level of DA and 5-HT of PD mice, likewise the MPTP-mediated increment in the ratio of (DOPAC+HVA)/DA and 5-HIAA/5-HT were decreased by CA, indicating that CA alleviated the MPTP-induced reduction in striatal DA and 5-HT, and contributed to the restoration of their metabolite level.

### 2.4. CA Suppressed Glial-Mediated Neuroinflammation Accompanied by an Increment in Striatal Neurotrophic Factors

**Figure 3:**
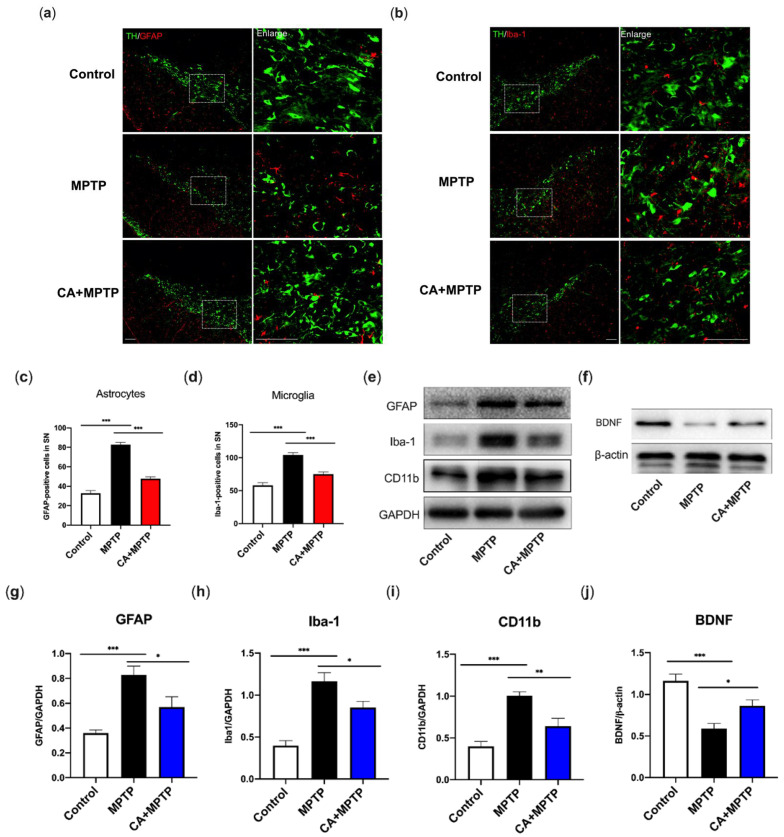
CA inhibited MPTP-induced microglial and astrocyte activation in SN and striatum, respectively, accompanied by the restoration of striatal BDNF. (**a**) Representative double-IF staining for TH (green) and GFAP (red) in SN. Scale bars: 100 µm. (**b**) Representative double-IF staining for TH (green) and Iba-1 (red) in SN. Scale bars: 100 µm. (**c**) Quantification for the number of GFAP-positive cells. (**d**) Quantification for the number of Iba-1-positive cells. (**e**,**f**) Representative bands of Western blotting for the expression of striatal GFAP, Iba-1, CD11b and BDNF. (**g**–**j**) Quantification for protein expression, band intensity normalized to GAPDH or β-actin. Statistical comparison by one-way ANOVA with Tukey’s post hoc test. Data represent means ± SEM. * *p* < 0.05, ** *p* < 0.01, *** *p* < 0.001. *n* = 4 for IF, *n* = 5 for Western blotting.

To investigate whether the neuroinflammation accompanied by glial activation occurs in PD mice, glial fibrillary acidic protein (GFAP) and ionized calcium binding adaptor molecule-1 (Iba-1), the markers of astrogliosis and microgliosis, were detected by double IF staining for TH and GFAP or Iba-1, respectively. Simultaneously, GFAP, Iba-1, cluster of differentiation molecule 11b (CD11b) (microglia marker) and BDNF in the striatum were examined by Western blotting to verify the changes in striatal protein and neurotrophics. The highest level of GFAP and Iba-1 in SN were observed in MPTP group along with the reduction in TH-positive cells (Figure 3a–d). CA-pretreated mice exhibited an inhibition of astrocyte and microglial activation shown as a decline in number of positive cells for GFAP and Iba-1, respectively (Figure 3a–d), as well as a higher level of TH-positive cells. Western blotting analysis confirmed the neuroinflammatory response induced by MPTP, as evidenced by the enhanced expression of striatal GFAP, Iba-1, as well as CD11b, another microglia-activation marker (Figure 3e,g–i). Of note, these increased proteins were reduced by CA (Figure 3e,g–i). We further explored the effects of CA on striatal BDNF, an essential neurotrophic factor for DA neuronal survival. As displayed in Figure 3f,j, a severe depletion of BDNF appeared in MPTP group; CA partially but significantly restored the BDNF level.

### 2.5. CA Restored the Protein Levels of IL-17, IFN-γ and TGF-β in the Serum, Striatum, Spleen and Solon of PD Mice

**Figure 4:**
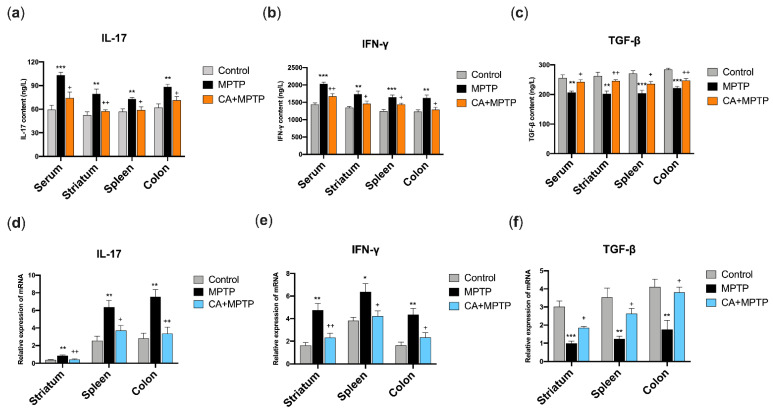
CA restored MPTP-mediated changes in IL-17, IFN-γ and TGF-β at the protein level and mRNA level in the serum, striatum, spleen, and colon. (**a**) The protein level of IL-17. (**b**) The protein level of IFN-γ. (**c**) The protein level of TGF-β. (**d**) The relative expression of IL-17 mRNA. (**e**) The relative expression of IFN-γ mRNA. (**f**) The relative expression of TGF-β mRNA. Statistical comparison by one-way ANOVA with Tukey’s post hoc test. Data represent means ± SEM. * *p*< 0.05, ** *p*< 0.01, *** *p* < 0.001, vs. Control group; + *p* < 0.05, ++ *p* < 0.01, vs. MPTP group. *n* = 4 for ELISA, *n* = 5 for qPCR.

In order to explore the influence of CA on the peripheral immune system, the protein expression of T helper 17 (Th17) cell-related cytokine (IL-17), T helper 1 (Th1)-related cytokine (IFN-γ) and regulatory T cells (Tregs)-related cytokine (TGF-β) in the serum, striatum, spleen and colon were determined by ELISA. As shown in Figure 4a–c, mice in MPTP group exhibited high levels of IL-17 and IFN-γ, and a low level of TGF-β in the serum, striatum, spleen and colon, respectively. Notably, CA pretreatment reduced the protein levels of IL-17 and IFN-γ, and increased TGF-β expression in the serum, striatum, spleen, and colon of PD mice.

### 2.6. CA Restored the mRNA Levels of IL-17, IFN-γ and TGF-β in the Striatum, Spleen and Colon of PD Mice

To further verify the ELISA results, the mRNA expressions of IL-17, IFN-γ and TGF-β of the striatum, spleen and colon were examined by quantitative PCR (qPCR). The primers of the cytokines are presented in Table 1. As expected, MPTP mice showed an enhancement of mRNA expression of IL-17 and IFN-γ, and a decrease in TGF-β in the striatum, spleen and colon (Figure 4d–e), respectively. These changes in mRNA expression were attenuated by CA, as a downregulation of IL-17 and IFN-γ mRNA, and an upregulation of TGF-β mRNA were observed in the striatum, spleen and colon of mice in CA+MPTP group (Figure 4d–e).

### 2.7. CA Modulated the Gene Expression of the Spleen in PD Mice Based on Transcriptome Analysis

The above results indicate that MPTP caused neuroinflammation and neurodegeneration, which was suppressed by CA markedly. In an attempt to explore the molecular mechanism role of the spleen and colon for the neuroprotection of CA in PD, the transcriptome profile of the spleen and colon was performed.

**Figure 5:**
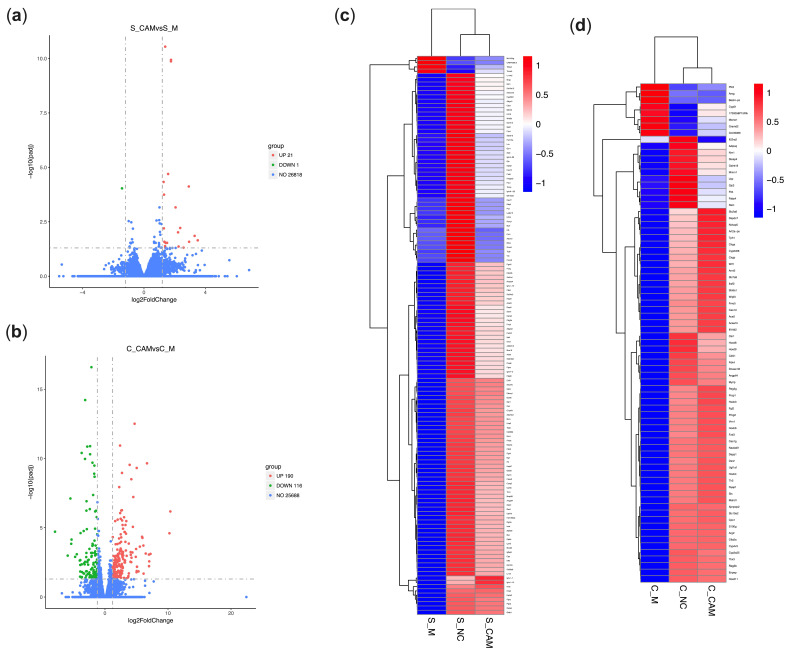
The influence of CA on splenic gene expression in PD mice based on RNA-Seq. (**a**) Volcano plot of splenic DEGs between the MPTP and CA+MPTP groups. (**b**) Volcano plot of colonic DEGs between the MPTP and CA+MPTP groups. The heatmaps showing the results of cluster analysis for DGEs between the control, MPTP and CA+MPTP groups in the spleen (**c**) and colon (**d**). CA pretreatment significantly restored the gene expression in the colon and spleen of MPTP mice; combining with the regulation of CA on IL-17, IFN-γ and TGF-β in PD mice, it was indicated that the neuroprotective effects of CA were closely related to the modulation of CA on peripheral immune system. Cut-off value of (|log_2_FC|) > 1.2 and padj < 0.05, *n* = 3 per group. S: spleen, C: colon. CAM: CA+MPTP group, M: MPTP group, NC: control group.

Differentially expressed genes (DEGs) were identified by the comparison between CA+MPTP and MPTP groups. The results show that a total of 22 DEGs in the spleen were expressed, of which 21 were upregulated and 1 was downregulated (Figure 5a). However, there were no significant differences of splenic gene expression between the CA+MPTP and control groups at the difference threshold of (|log_2_ Fold change (FC)|) > 1.2 and adjusted *p*-value (padj) < 0.05 (results not shown in figure). These results were also verified by the cluster analysis of splenic DGEs between the NC, MPTP and CA+MPTP groups (Figure 5c), as CA restored some splenic genes to normal levels in PD mice, suggesting that CA could improve neuronal survivals involving its regulation on splenic gene expression in MPTP mice.

**Figure 6:**
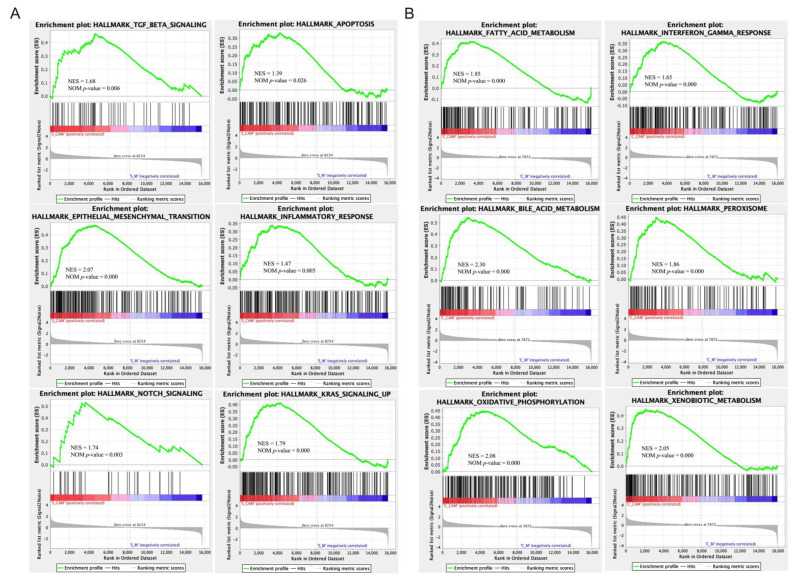
Enrichment plots from the gene set enrichment analysis (GSEA). (**A**) The significantly enriched hallmark terms associated with CA-mediated modulation on the spleen in PD mice. (**B**) The significantly enriched hallmark terms associated with CA-mediated modulation on the colon in PD mice. Cut-off value of |Normalized Enrichment Score (NES)| > 1.0 and nominal *p*-value < 0.05, *n* = 3 per group. S: spleen, C: colon. CAM: CA+MPTP group, M: MPTP group.

Next, the functional annotation of splenic DEGs between the CA+MPTP and MPTP groups was conducted using Gene Ontology (GO) and Kyoto Encyclopedia of Genes and Genomes (KEGG) enrichment with the significant cut-off value of padj < 0.05. The analysis of GO functional dissimilarity showed that the DEGs were mainly enriched in the cellular responses to dexamethasone and insulin stimulus, and the responses to dexamethasone, ketone, insulin, glucocorticoid and corticosteroid, as well as the immunoglobulin production and negative regulation of cytokine biosynthetic process (Table 2). The enrichKEGG analysis indicated that splenic DEGs were mainly involved in PPAR signaling pathway (Table 3). Gene Set Enrichment Analysis (GSEA) demonstrated that the expression of splenic genes induced by CA was positively enriched in epithelial mesenchymal transition, apoptosis, Kirsten rat sarcoma viral oncogene homolog (KRAS)-signaling, notch signaling, inflammatory response and TGF-β signaling (Figure 6a). The results reveal the molecular biological mechanisms for the regulation of CA on the spleen, which might be related to CA-mediated neuroprotection.

### 2.8. CA Modulated the Gene Expression of the Colon in PD Mice Based on Transcriptome Analysis

Inflammatory processes participate in the initiation and development of PD due to their effects on the brain–gut axis, including the immune system [19,20]. We further investigated the influence of CA on the colon in PD at the transcriptome level. As displayed in Figure 5b, a total of 306 colonic DEGs in PD mice were expressed with CA pretreatment, of which 190 were upregulated and 116 were downregulated. Notably, compared to the control group, only one downregulated DEG in the CA+MPTP group was obtained under the screening threshold of (|log_2_FC|) > 1.2 and padj < 0.05 (results not shown in the figures). Moreover, the results of cluster analysis for colonic DGEs between the NC, MPTP and CA+MPTP groups demonstrated that the MPTP-induced gene expression of the colon was significantly reversed by CA (Figure 5d). We thus inferred that CA pretreatment could modulate MPTP-induced gene expression in the colon, which was involved in the neuroprotection of CA.

GO enrichment results show that DEGs between MPTP and CA+MPTP mice were largely involved in the processes of fatty acid metabolism, organic acid biosynthesis, lipid catabolism and drug catabolism, as well as in the cellular response to xenobiotic stimulus, and the activity of monooxygenase and oxidoreductase; the rest of the enrichGO terms are displayed in Table 4. Furthermore, KEGG pathway annotations showed that colonic DEGs were mainly enriched in retinol metabolism, steroid hormone biosynthesis, chemical carcinogenesis, bile secretion and serotonergic synapse; the rest of involved pathways are displayed in Table 5. Additionally, GSEA enrichment plots indicated that the expression of colonic genes induced by CA were mainly enriched in fatty acid metabolism, IFN-γ response, oxidative phosphorylation, bile acid metabolism, peroxisome and xenobiotic metabolism (Figure 6b). These results provided biological molecular mechanisms in the CA-mediated communication between the brain and gut.

## 3. Discussion

Previous studies reported that the CNS inflammation could be regulated by systemic immunity targeting the spleen and gut, implicating the immunological communication between the brain and periphery [5,6]. In the current study, a neuroinflammatory response was observed in PD mice, as the glia population increased after being MPTP challenged, which was characterized by higher levels of CD11b, Iba-1 and GFAP, the hallmarks of activated microglia and astrocytes. In addition, MPTP induced an increment in IL-17 and IFN-γ, and decrease in the TGF-β of PD mice. Since IL-17, IFN-γ and TGF-β are well known as the markers of Th17, Th1 and Treg [21], respectively, it was suggested that the immunoreactions of peripheral organs participated in the neuroinflammatory and neurodegenerative progress of PD. Of note, Th1 and Th17, the CD4 effector T cells, have been considered as the potential contributors to neuroinflammation in PD [10], and microgliosis can exacerbate the deterioration of DA neuron via a proinflammatory mediator, such as IFN-γ released from Th1 [22]. Consistent with this, our data reveal gliosis-mediated neuroinflammation and enhancement in IL-17 and IFN-γ, whereas CA reduced their production, and prevented MPTP-mediated microgliosis as well as astrogliosis along with increased BDNF, the improvement in DA neuronal survival and motor function, suggesting that CA acted as an immunological mediator of the peripheral immune system and inhibited neuroinflammation in PD. Compelling evidence showed that the immunologic modulation of Tregs had an inhibitory effect on neuroinflammation leading to the reduction in the motor disorder of PD [22], and TGF-β released from Tregs acted against the MPP+-induced DA neuronal death via TβR-I on microglia [23,24,25], which also supported our results that CA upregulated TGF-β expression accompanied by the decrease in gliosis. Furthermore, a previous demonstration that CA could promote neuronal survivals and prevent LPS-induced neuroinflammation in vitro and vivo [16] is also consistent with our results. Accordingly, it is reasonable to infer that the neuroprotection of CA might be associated with the immunological modulation of CA on the peripheral immune system by targeting the spleen and colon.

CA is a diester of caffeic acid with L-tartaric acid (2,3-dicaffeoyl-L-tartaric acid), and a previous study has reported an inhibitory activity of caffeic acid against intestinal inflammation [26]. Additionally, gut inflammation may initiate and aggravate neuroinflammatory reaction in PD [20]. Our results demonstrated CA significantly alleviated gut inflammation and neuroinflammation in PD mice. Thus, targeting gut inflammation might be a promising therapy to inhibit neuroinflammation in PD and phenolic acid compounds with the basic structure of caffeic acid could be the potential candidates.

To further explore the prominent role of the spleen and gut in PD, and the regulatory effects of CA on them, we investigated transcriptome-level changes in the spleen and colon between the MPTP and CA+MPTP groups to provide a holistic view of genetic networks and demonstrate the molecular mechanism of the spleen and gut for the neuroprotection of CA on PD.

The heatmaps showed a remarkable gene expression change in the spleen and colon of PD mice, but CA pretreatment blocked these changes and restored some gene expression to normal levels, suggesting a favorable modulation of CA on the spleen and colon in PD. Notably, these genes might have a direct correlation with the self-function and self-homeostasis of organs and might be the potential targets for the regulation of CA on the spleen and colon in PD.

GO terms enriched with splenic and colonic DEGs were mainly involved in biological processes and cellular components, such as cellular responses to dexamethasone and insulin stimulus, and the responses to dexamethasone, ketone, insulin, glucocorticoid and corticosteroid, and the immunoglobulin production and negative regulation of cytokine biosynthetic process for the spleen, as well as the processes of fatty acid metabolism, organic acid biosynthesis, lipid catabolism and drug catabolism, cellular response to xenobiotic stimulus, and the activity of monooxygenase and oxidoreductase for colon. The KEGG analysis indicated peroxisome proliferator-activated receptors (PPAR) signal pathway for the spleen, and retinol metabolism, steroid hormone biosynthesis, chemical carcinogenesis, bile secretion and serotonergic synapse pathway for the colon. Moreover, GSEA identified some biological functions, including epithelial mesenchymal transition, apoptosis, KRAS-signaling, notch signaling, inflammatory response and TGF-β signaling, for the spleen, and fatty acid metabolism, IFN-γ response, oxidative phosphorylation, bile acid metabolism, peroxisome and xenobiotic metabolism for the colon. Thus, it is suggested that CA might influence the self-function and self-homeostasis of the spleen and colon to impact the neurological function in PD via these molecular biological processes and signaling pathways.

In this work, we observed that ERBB receptor feedback inhibitor 1 (ERRfi1), dual specificity phosphatase 1(Dusp1) and Phosphoenolpyruvate Carboxykinase (Pck1) were up regulated in the spleen. ERRfi1, also known as MIG-6 and RALT, is an indirect regulator for immunoreactions, which could drive epidermal growth factor receptors (EGFR) internalized and degraded due to its selective inhibition on EGFR [27,28], and multiple immune-related signal pathways can be activated by EGFR, including PI3K/AKT, STAT-1/STAT-3 and Raf/Ras/MEK/ERK [29]. Moreover, Dusp1 is described as an element of innate immunity to suppress the MAPK/ERK signals, which is essential for restraining the activation of JNK and p38 pathways to trigger anti-inflammatory responses [30,31]. Consistently, the results in this study showed that CA inhibited the IFN-γ-mediated inflammation in the spleen possibly via the related immunological pathways by upregulating ERRfi1 and Dusp1. In addition, PCK1 is a metabolic enzyme that is indispensable for gluconeogenesis [32,33], which is consistent with our results that the splenic PCK1 was upregulated and gluconeogenesis-related pathways, including PPAR signaling, are activated by CA. We inferred that CA exerted a neuroprotective effect on PD involving the brain–spleen axis by regulating these genes.

In PD, the imbalance of the microbiome–gut–brain axis plays a pivotal role [11], and gut microbial dysbiosis could conduce to the changes of metabolites, such as SCFAs, which might be one of the major mediators of neuroinflammation and gut inflammation [12,20]. In our previous work, we observed that CA showed a modulatory effect on gut microbiota and reduced gut inflammation [34], indicating that the gut is a crucial participant in PD. In the current study, transcriptome analysis was performed to examine the impact of CA on the gut in PD mice. PI3k serves as a pivotal part of the central pathway bearing cell proliferation, metabolism and cell survival, whose results alternate in the autophagy disruption in neurodegenerative diseases, including Alzheimer’s disease and PD [35]. In our study, gene PI3k of the colon was reversed to nearly to the normal level by CA, indicating that the regulation of CA on gene PI3k plays a key role in the gut function in PD. Furthermore, we found that the colonic dominant DEGs induced by CA in PD mice were mainly from cytochrome P450 (CYP) enzymes, and the inhibition and induction of CYP450 are the central mechanisms for pharmacokinetic interactions [36]. CYP450 pathway in vascular diseases provided anti-inflammatory oxylipins to prevent the inflammatory process [37]. We thus inferred that the anti-neuroinflammation of CA might be heavily involved in the inhibition of CA on gut inflammation via the regulation of CA on colonic CYPs. Notably, multiple pathways were activated by CA in the colon of PD mice, suggesting a modulatory role of CA on the gut–brain axis. For instance, retinol deficiency may lead to gut microbiota disorder that is proposed as a mediator of PD [38], and CA activated this pathway in PD via the regulation of related genes. In addition, steroid hormone is the initiator and maintainer for sexual differentiation and reproduction responsible for water and salt balance, metabolism and stress response [39]; in the current study, CA retained steroid hormone biosynthesis in the colon by regulating some gene expressions, including certain CYPs, which are the functional enzymes for biosynthesis of all steroid hormones. Moreover, the PPAR pathway, ligand-activated transcription factors, is capable of controlling energy homeostasis, its activation can boost fatty acid metabolism and enhance the glucose metabolism resulting from insulin sensitization [37,40]. Intriguingly, it was observed that the PPAR pathway was activated in both the spleen and colon in PD mice, suggesting that the CA-mediated PPAR pathway in the spleen and colon may play a key role in preventing the neurotoxicity of PD.

## 4. Materials and Methods

### 4.1. Materials

CA (≥98%) was purchased from Yiyan Bio-technology (Shanghai, China); its chemical structure is shown in Figure 1a. MPTP (M0896) was purchased from Sigma (Missouri, MO, USA). The primary antibodies used were mouse anti-TH (MAB318, Millipore, Burlington, MA, USA), rabbit anti- BDNF (ab108319, Abcam, UK), rabbit anti-GFAP (#80788, Cell Signaling, Beverly, MA, USA), rabbit anti-Iba-1 (#17198, Cell Signaling, Beverly, MA, USA), rabbit anti-CD11b (#17800, Cell Signaling, Beverly, MA, USA), mouse anti-β-actin (66009-1-Ig, Proteintech, Chicago, IL, USA) and rabbit anti-GAPDH (10494-1-AP, Proteintech, Chicago, IL, USA). The secondary antibodies used were FITC-conjugated goat anti-mouse IgG (A0568, Beyotime, Shanghai, China) and CY3-conjugated goat anti-rabbit IgG (A0516, Beyotime, Shanghai, China); goat anti-rabbit IgG (15015, Proteintech, Chicago, IL, USA) and goat anti-mouse IgG (15014, Proteintech, Chicago, IL, USA) conjugated to horseradish peroxidase (HRP). The materials for RNA-sequence are indicated in methods below.

### 4.2. Animals and Treatment

The male C57BL/6 mice used in this study were obtained from Beijing Weitonglihua Laboratory Animal Technology Institute (Shanghai, China). After acclimation under standard conditions at 20–22 °C on a 12–12 h light/dark cycle with food and water provided ad libitum, the mice (6–8 weeks old, 18 g ± 2 g) were randomly assigned to the control group, MPTP group and CA+MPTP group (*n* = 10 per group). For the CA treatment, mice were pretreated with CA (40 mg/kg) intragastrically daily for 12 days. For the MPTP injection, 2 h after CA administration, the mice received daily MPTP (30 mg/kg) injections intraperitoneally on the 8th day for 5 days. The mice in the control group were given the same volume of normal saline. After the last injection of MPTP, no further treatments were given to the animals for 7 days. The behavioral test was performed after 7 treatment-free days. Subsequently, the mice were sacrificed after being anesthetized with isoflurane (Yuyan, Shanghai, China); the serum, striatum, spleen and colon were harvested immediately, and the whole brain was obtained from the mice perfused transcardially with 0.01 M of phosphate-buffered saline (PBS) followed by 4% paraformaldehyde (PFA). This experimental timeline is illustrated in Figure 1b. All experimental procedures were approved by the Animal Ethics Committee of Jiangnan University (Wuxi, China).

### 4.3. Behavioral Tests

On the 5th day of the 7-day treatment-free period, the mice underwent behavioral training once per day for 3 days, and the formal behavioral experiments were conducted the next day. The behavioral test was carried out as previously described [41,42].

Pole test: a straight wooden pole with a diameter of 1 cm, a height of 50 and a spherical protuberance (diameter 2 cm) on the top was entangled with non-adhesive gauze. The mice were placed head-down on the top of the pole fixed in the home cage vertically, the total time of downward climbing from the top to the bottom of the pole was recorded for each animal. The test for each mouse was conducted three times at 10 min intervals, and the average time was obtained for the subsequent statistical analysis.

Traction test: the mice were placed on a straight and horizontal rope (diameter 5 mm) with the fore limbs gripping the rope; the placement of limbs was observed for 10 s and scored from 1 to 4. The scores were evaluated as: 1 for the mice gripping the rope with one front paw, 2 for the mice gripping the rope with both front paws, 3 for the mice gripping the rope with both front paws and 1 hind paw, and 4 for the mice gripping the rope with both front paws and both hind paws. The test for each mouse was repeated three times and the average score was obtained for statistical analyses.

### 4.4. Measurement of Neurotransmitters

Striatal neurotransmitters DA and 5-HT, and their metabolites including DOPAC, HVA and 5-HIAA were measured by HPLC (Waters 2475, Milford, MA, USA) as described previously (Ahmed et al., 2014; Rangasamy et al., 2019). The HPLC was equipped with a fluorescence detector and an Atlantis T3 column (150 mm × 4.6 mm, 5 μm, Waters); gradient elution was performed with the mobile phases composed of water, acetonitrile and 0.01 M PBS (pH 4.0). Striatum from half of the brain was homogenized in 0.1 M perchloric acid, and the resulting homogenates were centrifuged at 13,000× *g*, 4 °C for 15 min to collect the supernatant. A total of 25 μL supernatant of each sample were injected into the column. The standard solution was freshly prepared with DA, 5-HT and their metabolites DOPAC, HVA, 5-HIAA. Then, standard peaks were generated to calculate the concentrations of each neurotransmitter in each sample. Data were presented in ng/mL.

### 4.5. Immunofluorescence

The whole brain for frozen sectioning was post-fixed in 4% PFA at 4 °C for 24 h, then dehydrated in 20% and 30% sucrose successively at 4 °C based on the subsidence of the brain in the solution. Next, the dehydrated brain was embedded in an optimal cutting temperature compound (SAKURA, Torrance, CA, USA) for frozen sectioning. Each mouse brain was cryosectioned at 10μm by cryostat microtome (Leica CM1950, Wetzlar Germany) and the sections containing major part of substantia nigra pars compacta from bregma −2.92 mm to −3.52 mm were used for immunofluorescent staining.

Brain slices were subjected to antigen retrieval by being immersed in 0.01 M sodium citrate buffer (pH 6.0) (Solarbio, Beijing, China), and blocked with 5% goat serum (*v*/*v*)(Beyotime, Shanghai, China) diluted with PBS containing 0.2% Triton X-100 (*v*/*v*) (Beyotime, Shanghai, China) for 1 h at 37 °C. The slices were then incubated overnight at 4 °C with the primary antibodies against TH (1:1000), GFAP (1:2000) or Iba-1 (1:1000), and then incubated with the secondary antibodies conjugated to the FITC or Cy3 (1:1000) in the dark for 1 h at 37 °C. Finally, the brain slices were mounted with a mounting medium containing 4′,6-diamidino-2-phenylindole (DAPI) and imaged with a fluorescence microscope (ZEISS AXIO IMAGER, Oberkochen, Germany). The positive cells for TH, GFAP and Iba-1 were counted by a blind experimenter using Image J software (National Institutes of Health, Bethesda, MA, USA). A total of 6 brain sections containing major part of SN were quantified for each animal, and 5 animals in each group.

### 4.6. Western Blot Analysis

Striatum tissues were homogenized in ice-cold RIPA buffer (Beyotime, China) with an addition of a protease and phosphatase inhibitor cocktail (Solarbio, China), then centrifuged at 13,000 rpm, 4 °C for 10 min to collect supernatant. The concentration of proteins was determined using a BCA kit (BioSharp, Shanghai, China) following the manufacturer’s instructions. The supernatant samples were boiled at 95 °C for 10 min with loading buffer. A total of 30μg of total proteins from each sample were separated by electrophoresis in 10% sodium dodecyl sulfate-polyacrylamide gel electrophoresis (SDS-PAGE), then transferred onto polyvinylidene fluoride (PVDF) membranes (Millipore, Burlington, MA, USA). After being blocked in 5% BSA at room temperature for 2 h, the membranes were probed overnight at 4 °C with primary antibodies against TH (1:1000), BDNF (1:1000), CD11b (1:1000), GFAP (1:1000), Iba-1 (1:500), GAPDH (1:4000) and β-actin (1:2000), then incubated at room temperature for 2 h with HRP-conjugated secondary antibodies: goat anti-rabbit IgG (1:8000) and goat anti-mouse IgG (1:4000). Finally, the protein bands were exposed to be visualized using the super enhanced chemiluminescent (ECL; Millipore, Burlington, MA, USA) and imaged by a Gel Image System (Bio-Rad, Hercules, CA, USA). The protein content was normalized to GAPDH or β-Actin, and the grey value was analyzed by Image J software.

### 4.7. Enzyme-Linked Immunosorbent Assay (ELISA)

The content of IL-17, IFN-γ and TGF-β in the serum, striatum, spleen and colon was detected by a commercial ELISA kit (Mouse IL-17 Kit, Mouse IFN-γ Kit and TGF-β) (Nanjing Senbeijia Bioengineering Institute, China). All experimental procedures were conducted according to the manufacturers’ protocol. IL-17, IFN-γ and TGF-β concentration were expressed in ng/L protein.

### 4.8. qPCR for Cytokines IL-17, IFN-γ and TGF-β

Total RNA was extracted from the striatum, spleen and colon using the Trizol reagent (Invitrogen, Waltham, MA, USA) following the manufacturer’s protocol. Then, RNA samples were reverse transcribed into complementary DNA (cDNA) using the PrimeScript RT Master Mix reverse transcription kit (TaKaRa, Shiga, Japan). The mRNA expression was quantified by qPCR with SYBR Green Master Mix (Roche, Mannheim, Germany) and CFX96TM Real-Time System (Bio-Rad, Hercules, CA, USA). Relative gene expression was calculated by the ΔΔCt method, and Ct value was normalized to β-actin.

### 4.9. The Isolation of Total RNA

RNAs samples were isolated from the spleen and colon tissue using TRIzol Reagent (Invitrogen, Waltham, MA, USA) following the manufacturer’s protocol and treated with DNase using TURBO DNA-free kit (AM1907, Thermo Fisher Scientific (Waltham, MA, USA)). The quality and quantity of total RNAs were determined by the Agilent 2100 Bioanalyzer system. RNA samples whose RNA integrity numbers were greater than 8.0 were submitted to the following library preparation.

### 4.10. cDNA Library Construction and Sequencing

The transcriptome library was constructed using NEBNext^®^ Ultra^TM^ RNA Library Prep Kit (NEB, Ipswich, MA, USA) according to the manufacturer’s recommendations. The oligo(dT)-enriched mRNA was fragmented, and the interrupted RNA fragments were converted into the first cDNA strand using random hexamer primers and reverse transcriptase, followed by the synthesis of the second cDNA strand using DNA Polymerase I and RNase H. Subsequently, the double-stranded cDNAs were end-repaired and ligated to adaptors. Next, the size-selected cDNA was enriched by polymerase chain reaction (PCR), and the PCR products were purified by AMPure XP system to establish the final transcriptome library. After being assessed for quality by the Agilent Bioanalyzer 2100 system, the library was sequenced on an Illumina Novaseq platform (Illumina, San Diego, CA, USA). Each group consists of three biological replicates each with three technical replicates. The gene expression data were submitted to the National Center of Biotechnology Information (NCBI) Sequence Read Archive (SRA) repository (https://submit.ncbi.nlm.nih.gov/about/sra/, accessed on 20 October 2021) under accession numbers SRX12648145, SRX12648146, SRX12648147, SRX12639649, SRX12639650, SRX12639651, SRX12648142, SRX12648143, SRX12648144, SRX12639641, SRX12639642, SRX12639645.

### 4.11. Sequencing Data Analysis

DEGs were measured by the DESeq2 R package (1.16.1) with |log_2_FoldChange| (|log_2_FC|) > 1.2 and adjusted *p*-value (padj) < 0.05. The functional annotation of DEGs was performed with GO and pathway enrichment assessment; the functions enrichGO and enrichKEGG were analyzed using clusterProfiler package R under the R programing environment. GSEA was performed using the TCGA dataset with |NES| > 1.0 and nominal *p*-value < 0.05.

### 4.12. Statistical Analysis

Data are expressed as the mean ± SEM. Statistical analyses were conducted using GraphPad Prism Software 8.2.1. Differences were analyzed by one-way analysis of variance (ANOVA) with Tukey’s post hoc test for multiple groups comparisons. A *p*-value of <0.05 was set as the threshold for significance.

## 5. Conclusions

The present findings demonstrated that CA could prevent neuroinflammation and neurodegeneration via motor deficits, DA neuronal survivals in SN and glial reactions, along with the increased BDNF, DA and 5-HT of PD mice. The underlying mechanism might be associated with the modulatory role of CA on the immunological response and gene expression of the spleen and colon. This study provides the supportive evidence that the effects of CA on the spleen and colon may play a neuroprotective role in PD. However, further studies are needed to elucidate how a dysfunctional spleen and colon affect the brain–spleen and brain–gut axes in the pathology and development of PD.

## Figures and Tables

**Table 1:** Sequences of PCR primers.

Gene	Forward Primers	Reverse Primers
IL-17	5′-TGGACTCTGAGCCGCAATG-3′	5′-GGCGGACAATAGAGGAAACG-3′
IFN-γ	5′-CATAGATGTGGAAGAAAAGAG-3′	5′-AGAGTCTGAGGTAGAAAGAGATA-3′
TGF-β	5′-CGAAGCGGACTACTATGCTA-3′	5′-GAATGTCTGACGTATTGAAGAA-3′
β-actin	5′-CCTCTATGCCAACACAGT-3′	5′-AGCCACCAATCCACACAG-3′

**Table 2:** List of GO terms enriched with splenic DEGs (padj < 0.05).

Category	GO ID	Description	Gene Name	Count	Up	Down	padj
BP	GO:0042036	negative regulation of cytokine biosynthetic process	Errfi1↑Muc16↑	2	2	0	0.037678798
BP	GO:0051384	response to glucocorticoid	Errfi1↑Dusp1↑Pck1↑	3	3	0	0.037678798
BP	GO:0031960	response to corticosteroid	Errfi1↑Dusp1↑Pck1↑	3	3	0	0.037678798
BP	GO:0032869	cellular response to insulin stimulus	Pdk4↑Errfi1↑Pck1↑	3	3	0	0.037678798
BP	GO:1901654	response to ketone	Errfi1↑Dusp1↑Pck1↑	3	3	0	0.037678798
BP	GO:0071549	cellular response to dexamethasone stimulus	Errfi1↑Pck1↑	2	2	0	0.037678798
BP	GO:0071548	response to dexamethasone	Errfi1↑Pck1↑	2	2	0	0.046519989
BP	GO:0002377	immunoglobulin production	Igkv9-124↑Igkv14-100↑Igkv4-91↑	3	3	0	0.046927892
BP	GO:0032868	response to insulin	Pdk4↑Errfi1↑Pck1↑	3	3	0	0.046927892
↑ upregulation.							

**Table 3:** List of pathway enriched with splenic DEGs (padj < 0.05).

KEGG ID	Description	Gene Name	Count	Up	Down	padj
mmu03320	PPAR signaling pathway	Pck1↑Plin4↑	2	2	0	0.00949221
↑ upregulation.						

**Table 4:** List of GO terms enriched with colonic DEGs (padj < 0.05).

Category	GO ID	Description	Gene Name	Count	Up	Down	padj
BP	GO:0006805	xenobiotic metabolic process	Acaa1b↑Cyp2c66↑Ugt1a1↑Lpo↑Cyp2c65↑Cyp2c55↑Nceh1↑Ugt1a7c↑Cyp2d12↓Cyp2d9↓Srd5a2↓Cyp2c68↓Cyp2c69↓Cyp2f2↓	14	8	6	1.06 × 10^−8^
BP	GO:0042737	drug catabolic process	Cyp2c66↑Cyp2c65↑Akr1c18↑Cyp4b1↑Adh5↑Cyp2c55↑Cubn↑Aldh3b1↑Cyp2d12↓Cyp2d9↓Adh1↓Cyp2c68↓Nt5e↓Cyp2c69↓Cyp2f2↓	15	8	7	1.79 × 10^−7^
BP	GO:0042738	exogenous drug catabolic process	Cyp2c66↑Cyp2c65↑Cyp4b1↑Cyp2c55↑Cyp2d12↓Cyp2d9↓Cyp2c68↓Cyp2c69↓Cyp2f2↓	9	4	5	1.79 × 10^−7^
BP	GO:0006690	icosanoid metabolic process	Cyp2c66↑Cyp2c65↑Akr1c18↑Cyp2c55↑Tlr2↑Ggt1↑Cyp2d12↓Cyp2d9↓Pla2g4f↓Cyp2c68↓Pla2g5↓Cyp2c69↓Cyp2f2↓	13	6	7	1.79 × 10^−7^
BP	GO:0006631	fatty acid metabolic process	Acaa1b↑Ces1f↑Ppara↑Cyp2c66↑Lpin2↑Ces1d↑Pdk4↑Acsf2↑Cyp2c65↑Akr1c18↑Lpl↑Cyp2c55↑Slc27a4↑Ggt1↑Ehhadh↑Abhd3↑Adipoq↑Cyp2d12↓Cyp2d9↓Pla2g4f↓Cyp2c68↓Cyp2c69↓Cyp2f2↓	23	17	6	3.37 × 10^−7^
BP	GO:0016042	lipid catabolic process	Acaa1b↑Ces1f↑Lpin2↑Ces1d↑Akr1c18↑Lpl↑Ces1g↑Aspg↑Nceh1↑Slc27a4↑Ugt1a7c↑Ehhadh↑Abhd3↑Adipoq↑Pla2g4f↓Srd5a2↓Pla2g2a↓Pla2g5↓Ces3a↓Hexb↓	20	14	6	7.85 × 10^−7^
BP	GO:1901568	fatty acid derivative metabolic process	Cyp2c66↑Cyp2c65↑Akr1c18↑Cyp2c55↑Tlr2↑Ggt1↑Cyp2d12↓Cyp2d9↓Pla2g4f↓Cyp2c68↓Pla2g5↓Cyp2c69↓Cyp2f2↓	13	6	7	2.80 × 10^−6^
BP	GO:0046394	carboxylic acid biosynthetic process	Gapdh↑Hkdc1↑Ppara↑Pdk4↑Akr1c18↑Nags↑Lpl↑Malrd1↑Aldh1a1↑Ggt1↑Abhd3↑Ugdh↑Tkfc↑Gpd1↑Rdh16↓Eno3↓Pla2g4f↓Hif1a↓Hk2↓Rdh9↓Pla2g5↓	21	14	7	2.80 × 10^−6^
BP	GO:0016053	organic acid biosynthetic process	Gapdh↑Hkdc1↑Ppara↑Pdk4↑Akr1c18↑Nags↑Lpl↑Malrd1↑Aldh1a1↑Ggt1↑Abhd3↑Ugdh↑Tkfc↑Gpd1↑Rdh16↓Eno3↓Pla2g4f↓Hif1a↓Hk2↓Rdh9↓Pla2g5↓	21	14	7	2.80 × 10^−6^
BP	GO:0071466	cellular response to xenobiotic stimulus	Acaa1b↑Cyp2c66↑Ugt1a1↑Lpo↑Cyp2c65↑Cyp2c55↑Nceh1↑Ugt1a7c↑Cyp2d12↓Cyp2d9↓Srd5a2↓Cyp2c68↓Cyp2c69↓Cyp2f2↓	14	8	6	2.80 × 10^−6^
MF	GO:0016712	oxidoreductase activity, acting on paired donors, with incorporation or reduction of molecular oxygen, reduced flavin or flavoprotein as one donor, and incorporation of one atom of oxygen	Cyp2c66↑Cyp2c65↑Cyp2d26↑Cyp4b1↑Cyp3a44↑Cyp2c55↑Cyp2d12↓Cyp2d9↓Cyp2c68↓Cyp2c69↓Cyp2f2↓	11	6	5	2.77 × 10^−9^
MF	GO:0004497	monooxygenase activity	Tph1↑Fmo5↑Cyp2c66↑Cyp2c65↑Cyp2d26↑Akr1c18↑Cyp4b1↑Cyp3a44↑Cyp2c55↑Akr1c19↑Cyp2d12↓Cyp2d9↓Cyp2c68↓Cyp2c69↓Cyp2f2↓	15	10	5	2.02 × 10^−8^
MF	GO:0008395	steroid hydroxylase activity	Cyp2c66↑Cyp2c65↑Cyp3a44↑Cyp2c55↑Cyp2d12↓Cyp2d9↓Cyp2c68↓Cyp2c69↓Cyp2f2↓	9	4	5	8.37 × 10^−7^
MF	GO:0052689	carboxylic ester hydrolase activity	Ces1f↑Ces1d↑Ces2b↑Car1↑Lpl↑Ces1g↑Aspg↑Nceh1↑Abhd3↑Bche↑Pla2g4f↓Pla2g2a↓Pla2g5↓Ces3a↓	14	10	4	8.37 × 10^−7^
MF	GO:0016705	oxidoreductase activity, acting on paired donors, with incorporation or reduction of molecular oxygen	Tph1↑Fmo5↑Cyp2c66↑Cyp2c65↑Cyp2d26↑Akr1c18↑Cyp4b1↑Cyp3a44↑Cyp2c55↑Akr1c19↑Cyp2d12↓Cyp2d9↓Cyp2c68↓Cyp2c69↓Cyp2f2↓	15	10	5	2.51 × 10^−6^
MF	GO:0008392	arachidonic acid epoxygenase activity	Cyp2c66↑Cyp2c65↑Cyp2c55↑Cyp2c68↓Cyp2c69↓Cyp2f2↓	6	3	3	5.79 × 10^−6^
MF	GO:0008391	arachidonic acid monooxygenase activity	Cyp2c66↑Cyp2c65↑Cyp2c55↑Cyp2c68↓Cyp2c69↓Cyp2f2↓	6	3	3	1.12 × 10^−5^
MF	GO:0046906	tetrapyrrole binding	Cyp2c66↑Lpo↑Cyp2c65↑Cyp2d26↑Cyp4b1↑Cyp3a44↑Cyp2c55↑Cubn↑Cyp2d12↓Cyp2d9↓Cyp2c68↓Cyp2c69↓Cyp2f2↓	13	8	5	1.56 × 10^−5^
MF	GO:0005506	iron ion binding	Tph1↑Cyp2c66↑Cyp2c65↑Cyp2d26↑Cyp4b1↑Cyp3a44↑Cyp2c55↑Cyp2d12↓Cyp2d9↓Cyp2c68↓Nt5e↓Cyp2c69↓Cyp2f2↓	13	7	6	4.05 × 10^−5^
MF	GO:0020037	heme binding	Cyp2c66↑Lpo↑Cyp2c65↑Cyp2d26↑Cyp4b1↑Cyp3a44↑Cyp2c55↑Cyp2d12↓Cyp2d9↓Cyp2c68↓Cyp2c69↓Cyp2f2↓	12	7	5	4.27 × 10^−5^
↑upregulation							
↓downregulation							

**Table 5:** List of pathways enriched with colonic DEGs (padj < 0.05).

KEGG ID	Description	Gene Name	Count	Up	Down	padj
mmu00830	Retinol metabolism	Ugt2b5↑Cyp2c66↑Ugt1a1↑Cyp2c65↑Cyp3a44↑Aldh1a1↑Ugt2b36↑Adh5↑Cyp2c55↑Gm15368↑Ugt1a7c↑Rdh16↓Adh1↓Cyp2c68↓Rdh9↓	15	11	4	1.76 × 10^−10^
mmu00140	Steroid hormone biosynthesis	Ugt2b5↑Cyp2c66↑Ugt1a1↑Cyp2c65↑Cyp2d26↑Akr1c18↑Cyp3a44↑Ugt2b36↑Cyp2c55↑Gm15368↑Ugt1a7c↑Cyp2d12↓Cyp2d9↓Srd5a2↓Cyp2c68↓	15	11	4	1.99 × 10^−10^
mmu05204	Chemical carcinogenesis	Ugt2b5↑Cyp2c66↑Ugt1a1↑Cyp2c65↑Cyp3a44↑Ugt2b36↑Adh5↑Cyp2c55↑Gm15368↑Ugt1a7c↑Aldh3b1↑Adh1↓Cyp2c68↓	13	11	2	4.60 × 10^−7^
mmu00591	Linoleic acid metabolism	Cyp2c66↑Cyp2c65↑Cyp3a44↑Cyp2c55↑Pla2g4f↓Cyp2c68↓Pla2g2a↓Pla2g5↓	8	4	4	1.34 × 10^−5^
mmu04976	Bile secretion	Ugt2b5↑Ugt1a1↑Slc51a↑Slc10a2↑Sct↑Ugt2b36↑Gm15368↑Nceh1↑Ugt1a7c↑Adcy9↑	10	10	0	0.000109402
mmu00053	Ascorbate and aldarate metabolism	Ugt2b5↑Ugt1a1↑Ugt2b36↑Gm15368↑Ugt1a7c↑Ugdh↑	6	6	0	0.000126403
mmu00982	Drug metabolism - cytochrome P450	Ugt2b5↑Fmo5↑Ugt1a1↑Ugt2b36↑Adh5↑Gm15368↑Ugt1a7c↑Aldh3b1↑Adh1↓	9	8	1	0.000135848
mmu00980	Metabolism of xenobiotics by cytochrome P450	Ugt2b5↑Ugt1a1↑Ugt2b36↑Adh5↑Gm15368↑Ugt1a7c↑Aldh3b1↑Adh1↓Cyp2f2↓	9	7	2	0.000182884
mmu04726	Serotonergic synapse	Tph1↑Cyp2c66↑Cyp2c65↑Cyp2d26↑Slc18a1↑Cyp2c55↑Cyp2d12↓Cyp2d9↓Pla2g4f↓Cyp2c68↓Htr4↓	11	6	5	0.000408708
mmu00590	Arachidonic acid metabolism	Cyp2c66↑Cyp2c65↑Cyp2c55↑Ggt1↑Pla2g4f↓Cyp2c68↓Pla2g2a↓Pla2g5↓	8	4	4	0.001096684
mmu00040	Pentose and glucuronate interconversions	Ugt2b5↑Ugt1a1↑Ugt2b36↑Gm15368↑Ugt1a7c↑Ugdh↑	6	6	0	0.001096684
mmu00983	Drug metabolism - other enzymes	Ces1f↑Ugt2b5↑Ugt1a1↑Ces1d↑Ces2b↑Ugt2b36↑Gm15368↑Ugt1a7c↑Gm45727↑	9	9	0	0.0021122
mmu03320	PPAR signaling pathway	Acaa1b↑Ppara↑Lpl↑Angptl4↑Hmgcs2↑Slc27a4↑Ehhadh↑Adipoq↑	8	8	0	0.002234301
mmu00860	Porphyrin and chlorophyll metabolism	Ugt2b5↑Ugt1a1↑Ugt2b36↑Gm15368↑Ugt1a7c↑	5	5	0	0.008876674
mmu00010	Glycolysis / Gluconeogenesis	Hkdc1↑Adh5↑Pgm2↑Aldh3b1↑Eno3↓Adh1↓Hk2↓	7	4	3	0.008876674
mmu00592	alpha-Linolenic acid metabolism	Acaa1b↑Pla2g4f↓Pla2g2a↓Pla2g5↓	4	1	3	0.01005902
mmu00500	Starch and sucrose metabolism	Hkdc1↑Sis↑Pgm2↑Hk2↓	4	3	1	0.030913453
mmu00520	Amino sugar and nucleotide sugar metabolism	Hkdc1↑Pgm2↑Ugdh↑Hk2↓Hexb↓	5	3	2	0.036511025
mmu00052	Galactose metabolism	Hkdc1↑Sis↑Pgm2↑Hk2↓	4	3	1	0.041823524
↑upregulation						
↓downregulation						

## Data Availability

All sequence data in this study are available in the National Center of Biotechnology Information (https://www.ncbi.nlm.nih.gov/bioproject/, accessed on 20 October 2021) under Accession No. PRJNA771869 and PRJNA771189.
